# Surface toll-like receptor 9 on immune cells and its immunomodulatory effect

**DOI:** 10.3389/fimmu.2023.1259989

**Published:** 2023-09-01

**Authors:** Mengyuan Kou, Liying Wang

**Affiliations:** ^1^ Department of Immunology, College of Basic Medical Sciences, Jilin University, Changchun, Jilin, China; ^2^ Department of Molecular Biology, College of Basic Medical Sciences, Jilin University, Changchun, Jilin, China

**Keywords:** surface toll-like receptor 9, neutrophils, B cells, erythrocytes, immunomodulation

## Abstract

Toll like receptor 9 (TLR9) has been considered as a crucial intracellular pattern recognition receptor in the immune system, which can directly or indirectly mediate innate and adaptive immune responses by recognizing CpG DNA in endosomes to initiate its downstream signaling. However, TLR9 can also be expressed on the membrane surface of some immune and non-immune cells, called surface TLR9 (sTLR9), which covers the TLR9 and its immunomodulatory role with a mysterious veil. In this review, we mainly focus on the sTLR9 expressed on neutrophils, B cells and erythrocytes, and its immunomodulatory roles displayed alone or in coordination with endosomal TLR9 (eTLR9), providing a theoretical reference for the application of its modulators.

## 1 Introduction

Toll like receptor 9 (TLR9) is a member of the Toll-like receptor (TLR) family (1), which can be activated by the pathogen-derived non-methylated cytidine-phosphate-guanosine DNA (CpG DNA) or artificial synthetic non-methylated CpG containing oligodeoxynucleotides (CpG ODN) (2), and directly or indirectly initiate the immune response through its downstream signaling, thus resisting the pathogen invasion (3). TLR9 has long been considered to be an intracellular DNA sensor located in endolysosomes that is expected to prevent the extracellular DNA from activating TLR9 to mediate the initiation of an autoimmune response (4). However, with the deepening of studies, researchers found that TLR9 can also be expressed on the surface of cell membranes, such as neutrophils (5), B cells (6) and even erythrocytes (7), called surface TLR9 (sTLR9). According to studies, the activation of cellular immune responses can be initiated at these two TLR9 sites both inside and outside the cell. Moreover, the localization of TLR9 to the cell membrane contributes to the activation of endosomal TLR9 (eTLR9)-mediated signaling pathways (8). As a newly included immune cells, erythrocytes can mediate the activation of innate immune cells, such as macrophages, through sTLR9, and thus accelerate the clearance of itself in inflammatory state (9). Therefore, the presence of sTLR9 may benefit host responses in certain cell types and/or organs, and help determine whether the localization of TLR9 on the cell surface is essential for the TLR9-mediated immune response or a reserve pool for eTLR9. Here, the structural characteristics and trafficking of sTLR9, the relationship between sTLR9 and CpG DNA, and the immunomodulatory role of sTLR9 in immune cells are reviewed.

## 2 The structure and migration of sTLR9 and its relationship with CpG ODN

sTLR9 has been found on the membrane surface of a variety of immune cells, including neutrophils (5), B cells (6), splenic dendritic cells (10), splenic mononuclear cells (11), plasmacytoid dendritic cells (pDCs) (10), LPS-stimulated human peripheral blood mononuclear cells (12), and RAW 264.7 macrophages (11). Besides, it can also be expressed on the surface of erythrocytes and certain tumor cells (13–15). This section elaborates the structure and migration of sTLR9 and its relationship with CpG ODN.

### 2.1 The structure of sTLR9

The structure of sTLR9 is not particularly clear, and whether it is the same as eTLR9 is controversial. As is well known, eTLR9 belongs to a type I transmembrane protein, which is composed of extracellular, transmembrane and intracellular regions (16–18). The extracellular region of eTLR9 is located in the endosome and consists of 25 leucine-rich repeats (LRRs), whose N- and C-terminals are called TLR9-N and TLR9-C respectively. The TLR9-N and TLR9-C can be released by aspartate endonuclease that acts on the Z-loop domain between LLR14 and LLR15 in the middle of eTLR9 to form the TLR9-N+C complex, which is the active form of eTLR9 (10, 19, 20). However, the structure of sTLR9 is not well understood. A study has shown that a monoclonal antibody that recognizes eTLR9 do not bind to sTLR9 (10). An antibody 26C593 that binds to TLR9-N is completely unable to bind the sTLR9 on human peripheral neutrophils detected by flow cytometry, which may indicate that the sTLR9 on these neutrophils is not the active form of the TLR9-N+C complex (5). While, both anti-full-length TLR9 (fTLR9) and anti-TLR9-N antibodies can all recognize the sTLR9 on B cells, suggesting that fTLR9 and TLR9-N are present on B cells (21). For liver cancer cells, fTLR9 exists on the cell membrane, while TLR9 in enzymatically hydrolyzed form is mainly located in the cytoplasm (15). The J15A7 monoclonal antibody recognizing TLR9-N can detect sTLR9 on dendritic cells (DCs), but not sTLR9 on splenic neutrophils, indicating the sTLR9 conformation on DCs and neutrophils is different (10, 22). The impression given by these studies is that the structure of sTLR9 can be either a full-length inactive form or an enzymatically hydrolyzed active form, which may vary from cell to cell.

### 2.2 The trafficking of sTLR9

**Figure 1:**
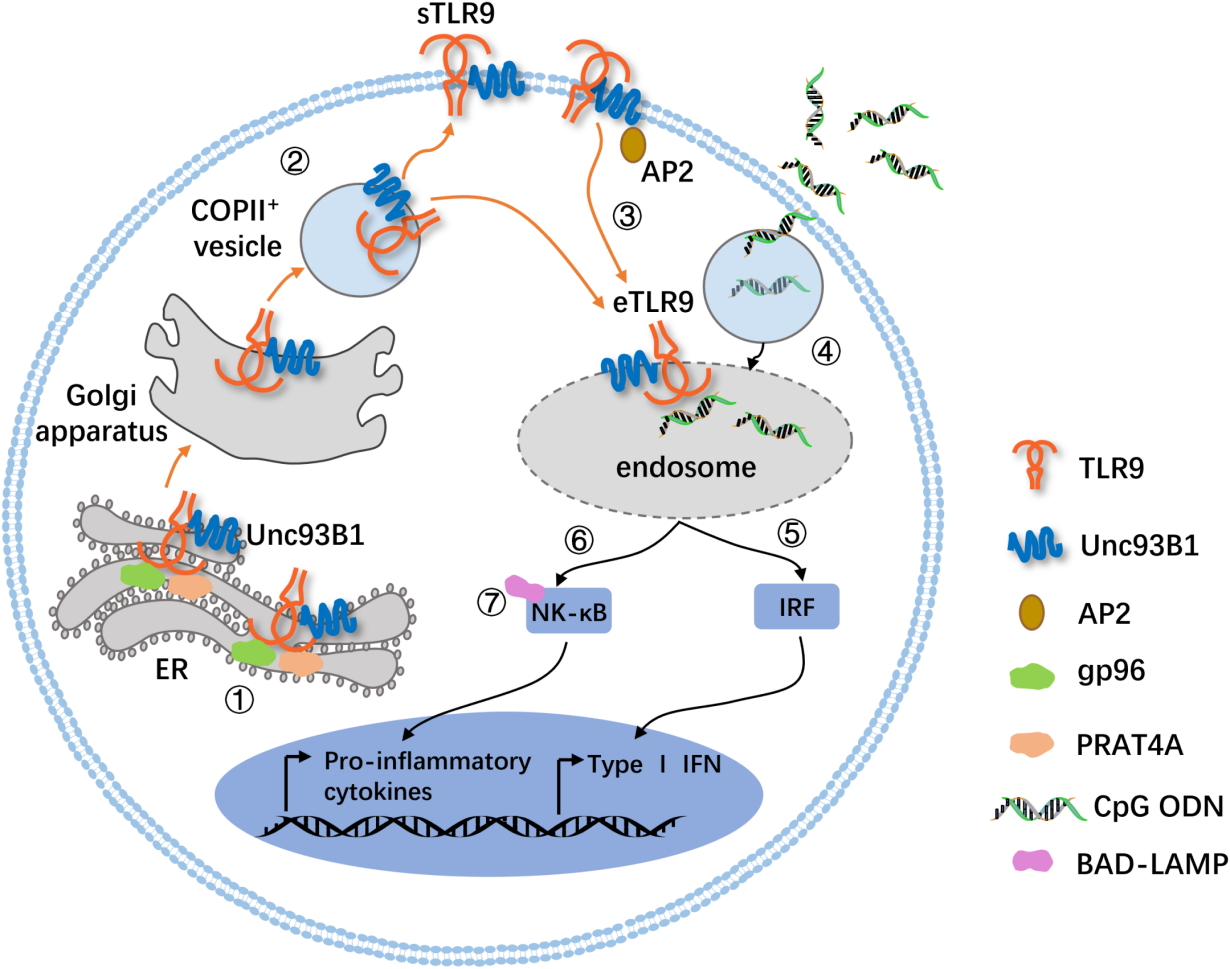
A mode diagram for the trafficking of sTLR9 and intracellular TLR9. 1) TLR9 is synthesized in the ER, and its proper folding require ER luminal chaperones gp96 and PRAT4A. 2) TLRO requires the ER membrane protein Unc93B1 to exit the ER. The TLR9 in the ER migrates outward via the Golgi secretion pathway and is loaded into COPII^+^ vesicles for further migration to the cell membrane surface or endolysosomal membrane. 3) Unc93B1 and TLR9 traffic together to the cell surface, and then Unc93B1 recruits AP2 (an adaptor protein complex that promotes endocytosis of cell surface molecules) to traffic TLR9 from the cell surface to endolysosomes. 4) In parallel, the TLR9 ligand, CpG ODN, is endocytosed in a clathrin-dependent manner and meets its cognate receptor. 5) and 6) Here, the pathway bifurcates. The migration of TLR9 to the IRF-SE induces the production of type I IFNs, and the localization to the NF-κB-SE induces the expression of proinflammatory cytokines. 7) In human pDCs, BAD-LAMP facilitates the trafficking of TLR9 from the IRF-SE to the NF-B-SE. g96, glycoprotein 96; PRAT4A, protein associated with TLR4A; AP2, adaptor protein 2; IRF-SE, IRF-signaling endosome; NF-κB-SE, NF-κB-signaling endosome; BAD-LAMP, brain and DC-associated LAMP-like molecule.

sTLR9 and its transport are related to the trafficking of intracellular TLR9. For the intracellular TLR9, it can locate on the membranes of either endoplasmic reticulum (ER) or endosome. For cells that express TLR9 constitutively, such as pDCs and B cells, TLR9 is mainly located on the ER membrane during homeostasis. However, TLR9 can migrate from the ER membrane to the endolysosomal membrane when stimulated by TLR9 ligands like CpG ODN to become eTLR9. This relocation process is dependent on several chaperone proteins, including uncoordinated 93 homolog B1 (Unc93B1) (23), glycoprotein 96 (gp96, a member of the ER-resident heat-shock protein 90 family, functions as a general chaperone for TLR9.) (24) and protein associated with TLR4 A (PRAT4A, also known as CNPY3, which is required for TLR9 folding.) (25). Unc93B1, a multipass transmembrane protein required for TLR9 function, is the most critical regulatory factor controlling trafficking of TLR9 from the ER to endolysosomes (23). Gp96 and PRAT4A also interact with TLR9 and seem to work together to coordinate the folding of TLR9 (**Figure 1**) (26). In the absence of gp96 and PRAT4A, TLR9 fails to exit the endoplasmic reticulum (24, 25). In fact, TLR9 can also migrate to the cell membrane surface by the assistance of Unc93B1 to become sTLR9 (10) and be proved to settle the surface depending on its transmembrane domain for mice (27). Unc93B1 was shown to be necessary for TLR9 to migrate from the ER to the surface of the cell membrane, since the transfection of only the TLR9 gene into TLR9-deficient cells would leave sTLR9 un-present (10). While Unc93B1 is an important chaperone for TLR9 migration to the endosome or cell surface, human TLR9 migration to the endosome has also been shown to be able to rely on the tyrosine motif of its cytoplasmic domain (28). This suggests that sTLR9 may be strictly dependent on Unc93B1 to migrate ER TLR9 to the cell surface, while eTLR9 may have more than one migration path, in other words, not only relying on Unc93B1. Besides, Unc93B1 can also enable the ER TLR9 to migrate to the Golgi apparatus when cells are infected by pathogenic microorganisms, and then the TLR9 is loaded into COPII^+^ (coat protein Complex II^+^) vesicles and transported to the cell membrane surface (**Figure 1**) (29, 30).

sTLR9 can also migrate from the cell surface to the endosome to become eTLR9 with the assistance of Unc93B1 and adaptor protein 2 (AP2) (**Figure 1**). It is found that Unc93B1 on the surface of the cell membrane recruits AP2 through its C-terminal YxxΦ motif and then forms a complex with clathrin to assist the transport of sTLR9 to the membrane of interferon regulatory factor (IRF)-signaling endosome (IRF-SE) or NF-κB-signaling endosome (NF-κB-SE). In the presence of TLR9 ligands such as CpG ODN, the migration of sTLR9 to IRF-SE is associated with the induction of type I interferon, while the migration of sTLR9 to NF-κB-SE is involved in the induction of inflammatory factors (**Figure 1**) (27, 31, 32). Moreover, in human pDCs, the brain and DC-associated LAMP-like molecule (BAD-LAMP), which is a member of the lysosome-associated membrane proteins (LAMPs), in conjunction with Unc93B1, promotes TLR9 trafficking from the IRF-SE to the NF-κB-SE, ultimately leading to the generation of pro-inflammatory cytokines (**Figure 1**) (33). These indicate that both sTLR9 and ER TLR9 can migrate to endosomes containing large amounts of TLR9 ligand CpG DNA. Although it is not clear how CpG DNA generates the signal to initiate TLR9 transport, it has been speculated that the imbalance of the amount of TLR9 in different sites may be the driving force to trigger the migration and relocation of TLR9 (34).

### 2.3 The relationship between sTLR9 and CpG ODN

Whether sTLR9 binds to the TLR9 ligand CpG ODN is still inconclusive to date, but several studies of sTLR9 expressed on neutrophils, B cells and erythrocytes have shown that sTLR9 can bind to CpG ODN and even initiate the TLR9 signaling independently of eTLR9. Studies have shown that sTLR9 on neutrophils could directly bind to CpG ODN and be activated (5). The co-incubation of FITC-labeled CpG ODN with membrane-ruptured neutrophils demonstrated its ability to enter cells and bind to eTLR9. Then, researchers incubated FITC-labeled CpG ODN directly with non-membrane ruptured neutrophils and found that the neutrophil had fluorescence on the its surface, indicating that CpG ODN binds to sTLR9 (5). When CpG ODN was fixed on a solid carrier so that it could not enter cells and bind to eTLR9, it was found that neutrophils could still be activated by CpG ODN, suggesting that TLR9 ligands may provide a rescue mechanism for neutrophil activation by triggering the sTLR9 pathway (5). Studies on the sTLR9 expressed on HEK293 transfected cells have shown that sTLR9 participated in intracellular signaling pathways in CpG ODN stimulation, involving in MyD88, IRAK, and TRAF6, indicating that TLR9 localized on the cell surface has biological activity (35). In addition, B cells and erythrocytes seem to have a similar phenomenon. After B cells were co-incubated with fluorescence-labeled CpG ODN, it was found by flow cytometry analysis that with the increase of CpG ODN concentration, the intensity of fluorescence signal detected on the cell surface gradually increased, suggesting that CpG ODN may bind to sTRL9 on B cells (6). DNA fragments containing CpG motifs from bacteria or mitochondria of damaged cells could bind to sTLR9 on nucleic acid-sensitive erythrocytes, thereby altering the erythrocyte morphology and activating the innate immune response (7, 9).

Thus, sTLR9 seems to have the ability to recognize and bind CpG ODN, but whether it can directly initiate signal transduction needs further study. Our study using mouse splenic B cells showed that CpG ODN made the sTLR9 on B cells migrate into the cell and enter the endolysosome to become eTLR9, participating in the signaling pathway of eTLR9. However, we do not prove whether the sTLR9 can bind to CpG ODN or not (36). Whether sTLR9 is able to bind to the TLR9 ligand is a worthy research topic, which is conducive to revealing the potential physiological significance of the appearance of TLR9 on the surface of cell membrane, and also helps to explain its possible role in autoimmune diseases.

## 3 The immunomodulatory effect of sTLR9

The immunomodulatory effect of sTLR9 on neutrophils and B cells have been studied in relatively depth. As an emerging sentinel in the human immune system, erythrocyte also express sTLR9, which can activate the innate immune response and has aroused a great interest in the academic circle.

### 3.1 Discovery of immunomodulatory effects of sTLR9

Over the past, TLR9 has been always known as an intracellular pattern recognition receptor (PRR). In 2004, it was reported that TLR9 can be expressed on the membrane surface of monocytes and B cells, called sTLR9. The study found that there were low levels of sTLR9 on newly isolated human monocytes, and the sTLR9 levels were significantly elevated after bacterial infection. The expression feature of sTLR9 in primary B lymphocytes is similar to that of monocytes (37). However, this study did not attract much attention. Then, in 2010, a paper published in Nature indicates that the damage-induced systemic inflammatory response is induced by the mitochondrial damage association molecular patterns (mtDAMPs) in bloodstream released from damaged cells, and the mitochondrial DNA (mtDNA) is the main (mtDAMPs) to activate TLR9 of neutrophils (38). Therefore, researchers proposed that mtDNA could also directly act on sTLR9 of neutrophils, thereby activating its downstream signaling pathway (39). In the following year of the above study, it was reported that C57BL/6 mice input adoptively with hematopoietic stem cells transfected with the transmembrane region mutants of TLR9 (TLR9^TM-MUT^) to overexpress the sTLR9, developed the fatal inflammation response (40). This is the first study to link the sTLR9 to immunomodulation.

By 2013, Lindau found that fresh human and mouse primary neutrophils could express sTLR9, and that this spontaneously expressed sTLR9 was functional (5). This study showed that TLR9 agonists and other stimulants could induce the expression of sTLR9 on neutrophils. CpG ODN that was cross-linked to solid carriers could still stimulate the activation of neutrophils, which is represented by up-regulation of the cytokine secretion and CD11b expression, indicating that sTLR9 on the neutrophil is able to play an induction role on its activation (5). Subsequent studies also supported this finding (5, 10, 22). It is not until now that the immunomodulatory function of sTLR9 has gradually attracted wide attention.

### 3.2 The immunomodulatory effect of sTLR9 on neutrophils

It has been reported that neutrophils are able to mediate innate immune responses via sTLR9 early in pathogen infection. It has been found that sTLR9-expressing neutrophils (sTLR9^+^ neutrophil) can sense the extracellular pathogen-derived TLR9 ligand CpG DNA when CpG DNA cannot enter the endosome or eTLR9 cannot be activated, and initiate the sTLR9-mediated signaling independently of eTLR9 (5). In addition, human neutrophils produce IL-8 in response to CpG ODN stimulation, even though they are pretreated with chloroquine to inhibit the endosomal acidification (5), suggesting that sTLR9 may play a role in mediating the response to CpG ODN.

**Figure 2:**
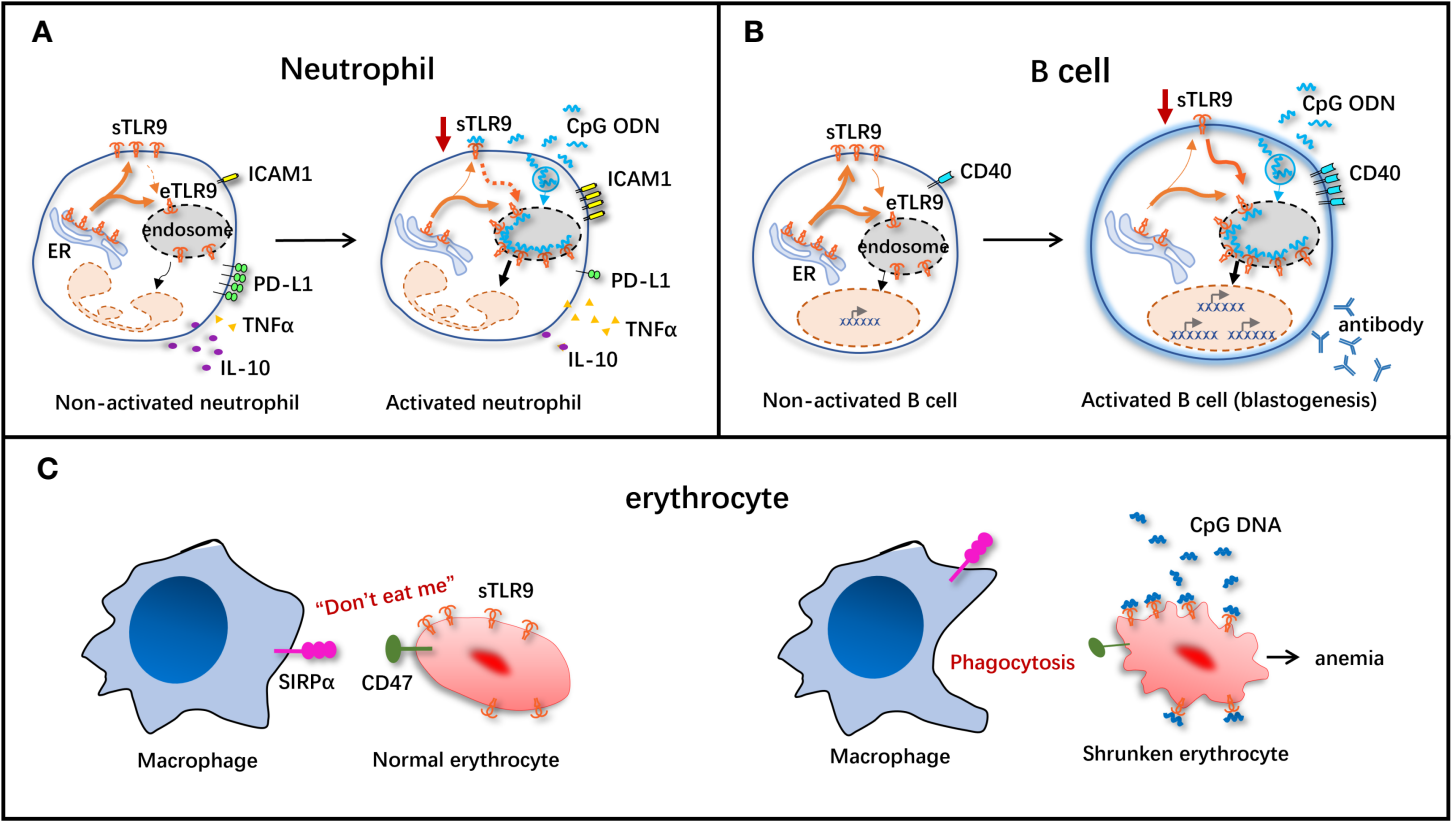
Schematic diagram of immunomodulatory effect of sTLR9 on immune cells. The immunomodulatory effect of sTLR9 on neutrophils **(A)**, B cells **(B)** and erythrocytes **(C)**. ICAMI, intercellular cell adhesion molecule 1; PD-LI, programmed death ligand 1; TNFα, tumor necrosis factor α: SIRPa, signal regulatory protein α. Dashed lines indicate that direct evidence is still absent, but present data support the hypothesis.

Some studies have suggested that sTLR9^+^ neutrophil may play a positive or negative immunomodulatory role in pathogen infection depending on its location or timing (**Figure 2A**). A study based on a mouse model of systemic inflammatory response syndrome (SIRS) induced by D-galactosamine (D-GAL) combined with CpG ODN showed that sTLR9^+^ neutrophil can produce both TNF-α and IL-10 in the inflammatory site. However, sTLR9^+^ neutrophil collected at 1 h after SIRS induction produced more IL-10 and less TNF-α than that collected at 6h, suggesting that sTLR9^+^ neutrophil at the initial stage of inflammation may be more inclined to negatively regulate the severe inflammatory reaction (41). In addition, intravenous transfusion of sTLR9^+^ neutrophil-rich peritoneal lavage cells (PLCs) collected at 1h but not at 6h after SIRS induction to the model mice could save the lives of the mice, which also demonstrated a negative immunomodulatory effect of sTLR9^+^ neutrophil at early stage of inflammation (41). Another study found that neutrophils in PLCs expressed more sTLR9 in the early stages of *E. coli* infection in mice, but decreased their expression levels in the later stage of the infection, further demonstrating the negative immunomodulatory properties of sTLR9^+^ neutrophil (42). Some studies have also pointed out that sTLR9^+^ neutrophil may be the culprit of aggravating the pathological change in the process of inflammation response. Pathogen infection, such as bacterial infection, can make the sTLR9^+^ neutrophil release a large number of pro-inflammatory cytokines, such as IL-6 and TNFα, thus aggravating the inflammatory response. In sepsis, the DNA or methylpeptide released by bacteria can bind to the sTLR9 on sTLR9^+^ neutrophil and activate the p38 mitogen-activated protein kinase (MAPK), leading to the development of acute lung injury characterized by protein-rich pulmonary edema and mass accumulation of neutrophils (38, 39). Despite these reports having contrary findings, the negative immunomodulatory effect of sTLR9 seems to be of more concern. In the study of neutrophils and pro-inflammatory factor IL-17 in SIRS model mice, we found that sTLR9^+^CD11b^+^ neutrophil did not promote the production of IL-17, but neutrophils that did not express sTLR9 increased the production of IL-17 (42). In tumors, sTLR9^+^ neutrophil is associated with the high expression of immunosuppressive molecules/cytokines such as programmed death ligand 1 (PD-L1) and IL-10 and the decreased expression of immunoactivators/molecules such as TNF-α and intercellular cell adhesion molecule 1 (ICAM1) (**Figure 2A**), and its proportion also increases with the progression of tumors (43), suggesting that sTLR9^+^ neutrophil has negative immunoregulatory properties. Down-regulating the level of sTLR9 on neutrophils in early tumors by using TLR9 agonist CpG ODN can inhibit the tumor growth (43), which also supports that sTLR9^+^ neutrophil plays a negative immunoregulatory role.

In summary, the presence of sTLR9 on the surface of neutrophils, whether inflammation or tumor, seems to serve as a molecular marker to determine whether it plays a positive or negative immunomodulatory role, while also giving sTLR9 the trait of an immune checkpoint molecule. TLR9 may play the dual role of immune checkpoint or immune activation through selective migration, and become a unique and rapid activity regulation mode of innate immune cells such as neutrophils.

### 3.3 Immunomodulatory effects of sTLR9 on B cells

Recent research has discovered that TLR9 is expressed on B cell surface, and is involved in B cell activation (6, 44, 45). According to research reports, compared with healthy controls, sTLR9 expression significantly increases in the peripheral blood B cells of septic patients (17). Activation of sTLR9 can suppress B cell proliferation and CD25 expression induced by CpG ODN alone or in combination with anti-IgM antibody (6). In the case of anti-IgM antibody-mediated B cell receptor (BCR), sTLR9 transfers to the lipid raft of B cells, indicating that sTLR9 may be delocalized during the B cell activation process, and that such re-localization may be at least partially necessary for the response of B cells to BCR activation (6).

There is also research suggesting that, sTLR9 may be a kind of negative regulatory factor for resting state B cells to antagonize eTLR9 activation, when B cells are activated via the eTLR9 signal, sTLR9 can assist eTLR9 in exerting its function (**Figure 2B**). Moreover, it is found in one study conducting antibody feeding experiment, under the stimulation of vaccine plus CpG ODN, the sTLR9 on B cells is internalized into cells and co-localized with the endosomal membrane protein, accompanied by the decreased sTLR9 level in B cells and the activation of B cells (enlarged volume and increased expression of CD40) (**Figure 2B**), revealing that sTLR9 is internalized into eTLR9 under the action of the TLR9 agonist (36). These data suggest that, one of the mechanisms of CpG ODN-mediated B cell activation may at least partially exist in its role in inducing sTLR9 re-localization in B cells, providing the disproof that sTLR9 may be a negative regulator for the TLR9-mediated excessive B cell activation.

### 3.4 Immunomodulatory effects of sTLR9 on erythrocytes

It is stated in the latest research that, erythrocytes can not only function to transport oxygen, but also play a role of immune sentinel, which can timely perceive the pathogens or injury in the body and exert the immune cell functions. Interestingly, the nucleic acid sensing receptor TLR9 is expressed on erythrocyte surface, which is associated with the activation of innate immunity and accelerated clearance of erythrocytes in the inflammatory state (**Figure 2C**).

Both pathogens invading human body and the injured cells can release CpG DNA, and thus the free CpG DNA level in the body elevates, which is dramatically linked to the occurrence of malignant tumors, autoimmune diseases and sepsis (38, 46–48). In 2018, Mangalmurti’s group demonstrated that, erythrocytes were necessary for clearing CpG DNA and alleviating lung tissue injury. As discovered in research, in the case of inflammation in human body, erythrocytes can scavenge the free CpG DNA, thus mitigating tissue injury (7). sTLR9 is expressed on the surfaces of both human and murine erythrocytes, and can bind to plenty of free CpG DNA (49). After binding to CpG DNA, the molecular conformation of CD47 (a kind of “don’t eat me” signal molecule) on erythrocyte surface is changed, which loses its original “don’t eat me” function. When such erythrocytes pass through the reticuloendothelial system including the liver and the spleen, they are rapidly recognized by signal regulatory protein α (SIRPα) expressed on macrophages and then phagocytized by macrophages (**Figure 2C**). Therefore, erythrocytes eliminate the TLR9 agonist CpG DNA in a self-suicidal manner. This suppresses the excessive inflammatory response, and induces the occurrence of anemia (9, 50). Moreover, this study also first explains the pathogenesis of acute severe inflammation usually accompanied by anemia from the erythrocyte sTLR9 perspective. Furthermore, researchers inject CpG DNA-binding erythrocytes into mice, inducing systemic inflammatory response in the mice. At 6 h after injection, the IFNγ and IL-6 levels in mouse plasma dramatically increase, revealing that erythrocytes depend on sTLR9 to scavenge CpG DNA while assisting in initiating the innate immune response (9). Even though, the erythrocytes that can express sTLR9 just account for a small proportion, and their roles in immunomodulation remain further investigations.

## 4 Possible mechanism of sTLR9 in exerting the negative immunomodulatory effects

As mentioned above, sTLR9 seems to exert the negative immunomodulatory function in neutrophils and B cells, consequently, down-regulating sTLR9 contributes to the activation of cells. Both sTLR9 and eTLR9 are mobile (51), and their migration to the endosome is a prerequisite for activation of TLR9-related signaling pathways (52). We find that sTLR9 on neutrophils and B cells can be down-regulated after CpG 5805 treatment (**Figures 2A, B**), then, if the decreased expression of sTLR9 is the consequence of sTLR9 transfer into the endosome? Through tracking the transfer path of sTLR9, it is found that CpG 5805 can promote the transfer of B cell sTLR9 into the cells, and finally into the endosome, demonstrating that the down-regulated sTLR9 on cell membrane surface has actually effectively entered the endosome. Coincidentally, for the B cells whose sTLR9 enter the endosome, they have apparently enlarged their volume (**Figure 2B**), while the increase of B cell size is an important manifestation of B cells, demonstrating that sTLR9 entering the endosome promoted cell activation (36). If so, reducing sTLR9 expression on the cell surface may promote cellular immunity via two manners. One is removing the suppression of sTLR9, which we speculate is a checkpoint molecule of innate immune response. The other one is promoting eTLR9 activation, aiming to increase the chance of TLR9 meeting its ligand (**Figures 2A, B**). After CpG 5805 induction, sTLR9 expression decreased on cells, while eTLR9 expression increased. The increased expression of eTLR9 indicates that TLR9 activation is enhanced by TLR9 agonist through the TLR9 signaling pathway. Cells that expressed less sTLR9 and more eTLR9 can produce more downstream cytokines of the TLR9 signaling pathway (**Figures 2A, B**).

Regarding the role of CpG ODN in promoting the transfer of sTLR9 into the endosome, some research suggests that it may be related to chaperonins Unc93B1 and AP2 (29). The hypothesis is based on one of our previous studies, which suggests that CCT ODN may interfere with the Unc93B1-mediated TLR9 transport into the CpG ODN-induced human pDC cell line CAL-1 (53). Moreover, such hypothesis also supported by the evidence that sTLR9 is expressed in the Unc93B1 gene transfection-induced sTLR9-negative cells (10).

## 5 Conclusion

The possibility that TLR9 may occur on the cell membrane and act as stable and functional receptor seems particularly interesting. Already, investigations reveal that localized in such a way, TLR9 may become disease conducive or act as salutary immune sensors. Consequently, TLR9 present on the plasma membrane may serve as therapeutic targets for functional monoclonal antibodies and might account for the progression of new therapeutical approaches towards rare human diseases that are difficult to treat. Discovering pathways originating from the cell surface TLR9 may uncover new functions of endosomal TLR9, enhance our comprehension of the triggering of inflammation or cancer diseases, and potentially expose previously unknown specific ligands for the sTLR9 receptor.

## Author contributions

MK: Investigation, Writing – original draft. LW: Funding acquisition, Writing – review & editing. All authors contributed to the article and approved the submitted version.
